# Factors Influencing Quality of Life in Early Postpartum Women

**DOI:** 10.3390/ijerph18062988

**Published:** 2021-03-14

**Authors:** Yu-Jeong Jeong, Ju-Hee Nho, Hye Young Kim, Ji Young Kim

**Affiliations:** 1 Department of Nursing, Presbyterian Medical Center, 365 Seowon-ro, Wansan-gu, Jeonju, Jeollabuk do 54987, Korea; yjjung00310@daum.net; 2 College of Nursing, Jeonbuk National University, 567 Baekje-daero, Deokjin-gu, Jeonju, Jeollabuk do 54896, Korea; tcellkim@jbnu.ac.kr (H.Y.K.); kimjjy@jbnu.ac.kr (J.Y.K.)

**Keywords:** postpartum fatigue, postpartum depression, marital intimacy, breastfeeding adaptation, quality of life

## Abstract

Postpartum women experience various changes in their physical and psychological health and in their relationships with their spouse and newborn. This study aimed to identify and evaluate the factors that affect the quality of life (QoL) of women within six weeks after childbirth. A prospective, cross-sectional correlational study was used. A convenience sample of 179 postpartum women was recruited from four postpartum care centers in South Korea. Participants completed structured questionnaires on postpartum fatigue, postpartum depression, marital intimacy, breastfeeding adaptation, and quality of life. Marital intimacy (β = 0.466, *p* < 0.001) was the most influencing factor on the QoL of women during the postpartum period. In descending order, postpartum fatigue (β = −0.192, *p* = 0.001), postpartum depression (β = −0.190, *p* = 0.001), breastfeeding adaptation (β = 0.163, *p* = 0.002), and occupation (β = 0.163, *p* = 0.004) all had a significant influence on QoL (F = 32.09, *p* < 0.001), and the overall explanatory power was 63.6%. It is necessary to assess and consider the physical, psychological, relational, and demographic factors of women during the early postpartum period. Comprehensive interventions need to be developed to improve the QoL of women during the postpartum period.

## 1. Introduction

Quality of life (QoL) is a multidimensional concept that is affected by physical, psychological, relational, and social well-being. Therefore, to understand the QoL of patients, practitioners must consider the various aspects involved [1]. During the postpartum period, the QoL of women should reflect their perception and satisfaction with their health concerns within their cultural context [2]. For new mothers who have to take care of themselves, childbirth and the many difficulties in caring for the baby have a negative impact on their QoL [3]. Additionally, the role and responsibility of mothers in caring for their newborn baby make a major difference to the QoL of women during this period [4]. According to a systematic review, clinical factors, such as delivering by cesarean section, are associated with QoL and health status [5]. Needing intensive care and having a newborn hospitalized are associated with poor QoL [6]. Postpartum women report a series of physical symptoms, such as fatigue, back pain, perineal pain, dyspareunia, hemorrhoids, and urinary incontinence [5,7], which are associated with QoL [6]. Postpartum fatigue is particularly common among new mothers [8], reported as the highest on the tenth day after childbirth (38.8%), and then gradually decreasing at one and three months (27.1% and 11.4%, respectively) [9].

Psychological changes also occur, such as an increased risk for depressive disorders [10]. A recent meta-analysis reported that postpartum depression affects approximately 17% of healthy women [11]. Depressive symptoms are detrimental to the health and QoL of both baby and mother [12].

Postpartum women likewise experience relationship changes with their spouses as a result of childbirth. In an Australian study, numerous factors are identified as affecting intimate relationships, including tiredness, changing lifestyles, and body image issues, leading to changes in intimacy [13]. Marital intimacy affects QoL; good marital intimacy lowers depression and anxiety and improves QoL in postpartum women [14,15]. 

Women during the postpartum period must adapt to their new role as a mother, which mainly requires breastfeeding and caring for the baby [16]. Breastfeeding adaptation refers to mothers and babies being adapted and educated to the changes during breastfeeding, which promote integration and sustain the practice of breastfeeding for a long time [17]. Successful breastfeeding provides many health benefits for both mother and baby, such as decreased postpartum depression, the anxiolytic effects of oxytocin, release of prolactin, emotional involvement, mother–child bonding, and healthy growth and development of the child [18,19].

Given the effects on QoL by various factors, health care research needs to comprehensively identify the factors that affect the QoL of postpartum women as they experience and adapt to various changes. However, it is difficult to find a study on how various factors, including depression and fatigue, affect the QoL in postpartum women. In addition, it is necessary to determine the degree of influence of these factors. Therefore, this study aimed to identify the variables that influence women’s QoL after childbirth, including the physical, psychological, and relational aspects, as well as adaptation to breastfeeding and the new role as a mother.

## 2. Materials and Methods

### 2.1. Design and Participants

This study used a cross-sectional correlational design. Between 20 June and 30 September 2019, participants were recruited via convenience sampling from postpartum care centers in Jeonbuk, South Korea. The eligibility criteria were as follows: (1) aged 20 years or older, (2) healthy women who delivered at 38 to 42 weeks and within six weeks, (3) breastfeeding women, (4) women with a spouse, (5) no obstetrical complication related to pregnancy, childbirth, and baby, (6) no medical history (e.g., thyroid disease, depression). Among 183 women recruited to participate in this study, 181 (98.9%) provided their written informed consent. Two participants were excluded for turning in incomplete questionnaires. Finally, 179 women (97.8%) were included in this study. 

Data were collected using structured questionnaires and face-to-face contact. The Institutional Review Board of Jeonbuk National University approved this study, and all participants provided informed consent. The study was conducted in accordance with the ethical standards of the institution’s Committee on Human Experimentation and the Helsinki Declaration of 1975.

### 2.2. Measures

#### 2.2.1. Maternal Postpartum Quality of Life (MAPP-QOL)

QoL was measured using MAPP-QOL [2], for which the Korean version has well-established reliability and validity [14]. The MAPP-QOL has 40 items and two parts (satisfaction and importance), and the scores are calculated by weighting each satisfaction response with its paired importance response. The QoL score ranges from 0 to 30, with higher scores indicating higher QoL. Cronbach’s alpha was 0.96 in the original study [2] and 0.96 in our study.

#### 2.2.2. Fatigue Continuum Form (FCF) 

The Korean version FCF was used to assess the participants’ fatigue [20]. The FCF includes 30 items, each rated from 1 to 4. Higher scores indicate higher fatigue. Cronbach’s alpha was 0.92 in the original study [21] and 0.92 in the current study.

#### 2.2.3. Edinburgh Postnatal Depression Scale (EPDS) 

The EPDS is a set of 10 screening questions that can be used to assess postpartum depression [22]. Responses are scored 0, 1, 2, and 3 depending on the seriousness of the symptom, and higher scores indicate higher degrees of depression. Cronbach’s alpha was 0.87 in the original study [22] and 0.77 in our study. The Korean version of EPDS has well-established reliability and validity [23].

#### 2.2.4. Marital Intimacy (MI)

The marital intimacy scale developed by Lee [24] was used to assess the marital intimacy levels of postpartum women. The scale consists of three domains of marital intimacy related to cognition, emotion, and sexuality; it includes 15 items rated on a five-point Likert scale. Total scores range from 15 to 75 points, with higher scores indicating greater marital intimacy. Cronbach’s alpha was 0.90 in the original study [24] and 0.92 in our study.

#### 2.2.5. Breastfeeding Adaptation Scale (BFAS) 

The BFAS developed for Korean postpartum women was used to measure breastfeeding adaptation [17]. This scale includes 27 items rated on a five-point Likert scale from 1 (disagree) to 5 (absolutely agree). Higher scores represent higher levels of adaptation to breastfeeding. Cronbach’s alpha was 0.82 in the original study [17] and 0.89 in our study.

#### 2.2.6. General Characteristics

Demographic and clinical characteristics were also obtained, including age, age of spouse, education level, job, religion, pregnancy type, delivery type, baby’s gender and birth order, baby care assistant, and monthly household income.

### 2.3. Analysis

The collected data were analyzed using the SPSS 26.0 (IBM SPSS Statistics for Windows; IBM Corp., Armonk, NY, USA). Postpartum fatigue, postpartum depression, marital intimacy, breastfeeding adaptation, QoL, and general characteristics were analyzed to obtain descriptive statistics. We used independent *t*-test and one-way analysis of variance to identify QoL according to the general characteristics. The correlation analysis of postpartum fatigue, postpartum depression, postpartum marital intimacy, breastfeeding adaptation, and QoL of the participants was calculated by Pearson correlation coefficient. The hierarchical regression method was performed to identify the factors influencing the QoL of women. Two-tailed *p* < 0.05 was considered statistically significant.

## 3. Results

### 3.1. General Characteristics of Participants and Score of Variables

Table 1 and Table 2 summarize the participants’ general characteristics and scores of variables. The mean age of the women and their spouse was 32.9 ± 4.3 and 35.7 ± 5.0 years, respectively. Most of the women (91.6%) had a natural pregnancy, and 39.1% of the women had vaginal delivery. Additionally, all variables are considered normally distributed.

### 3.2. Quality of Life According to General Characteristics

Table 3 lists the QoL according to the participants’ general characteristics. Women under the age of 35 years (t = 2.51, *p* = 0.013), a job (t = 3.83, *p* < 0.001), a baby care assistant (t = 2.53, *p* = 0.012), and higher family income (F = 4.65, *p* = 0.004) had higher QoL scores than their counterparts who were older than 35 years, without a job, had no baby care assistant, and had a lower family income.

### 3.3. Postpartum Fatigue, Postpartum Depression, Marital Intimacy, Breastfeeding Adaptation, and Quality of Life 

The QoL scores showed a significant correlation with the scores for postpartum fatigue (r = −0.48, *p* < 0.001), postpartum depression (r = −0.53, *p* < 0.001), marital intimacy (r = 0.67, *p* < 0.001), and breastfeeding adaptation (r = 0.49, *p* < 0.001) (Table 4).

### 3.4. Factors Influencing Quality of Life in Postpartum Women 

Hierarchical regression analysis, including the statistically significant variables evaluated in the univariate analyses, was performed to examine the factors that influenced the participants’ QoL (Table 5). Marital intimacy was the most significant factor affecting maternal QoL (β = 0.466, *p* < 0.001), followed by postpartum fatigue (β = −0.192, *p* = 0.001), postpartum depression (β = −0.190, *p* = 0.001), breastfeeding adaptation (β = 0.163, *p* = 0.002), and occupation (β = 0.163, *p* = 0.004; F = 32.09, adjusted R^2^ = 0.636, *p* < 0.001).

## 4. Discussion

The hierarchical regression analysis, conducted to identify the factors affecting the QoL of women during the postpartum period, revealed that marital intimacy had the greatest effect, followed by postpartum fatigue, postpartum depression, breastfeeding adaptation, and occupation, in this order. In other words, marital intimacy has graver implications than postpartum fatigue or depression on the QoL of women during the postpartum period. Indeed, spousal support increases the QoL of women during the postpartum period [25], whereas its absence increases the risk for severe postpartum depression [26]. The loss of libido, imbalances in sexual desire between partners, and lifestyle changes (e.g., no longer being at work, decreased economic status, reduced social contact) have been reported as contributing factors to decreased intimacy between postpartum women and their partners [13]. Additionally, communication difficulty, unrealistic expectations from marriage and the spouse, and lack of expression of affection may negatively affect marital stability [27,28]. Cognitive-behavioral interventions (including promoting communication, problem solving, self-disclosure, and empathic response skills) and sex education can improve marital intimacy [29]. Therefore, healthcare providers should consider cognitive-behavioral interventions that help enhance marital intimacy in postpartum couples. The instrument used in the present study to measure marital intimacy consisted of three sub-domains: cognitive, emotional, and sexual. The participants were women within six weeks postpartum, and it is said that one can resume sexual intercourse within six weeks postpartum [30]; however, we did not confirm the actual resumption of sexual intercourse in the present study. This may have influenced the score of marital intimacy including the sexual domain. It is expected in future studies to consider the participants’ actual resumption period and assessment including various areas of marital intimacy. 

After giving birth, women feel tired throughout the postpartum period, especially for those who have difficulty adapting to the changed environment [31]. Postpartum fatigue is a common symptom that occurs in 27% to 39% of new mothers within one month after childbirth [9], and is especially the highest in the early postpartum period [32]. Women feel more easily fatigued amid changes in their emotional mood, such as the case in postpartum depression; moreover, the level of fatigue depends on the baby’s sleep or lactation status [9,33]. Our study showed postpartum fatigue as the second factor affecting QoL, consistent with previous finding that postpartum fatigue reduced the mother’s comfort, breast milk production, and QoL [34]. Postpartum fatigue also negatively affects the QoL of not only women but also their families [35]. Therefore, health care providers should assess and manage the postpartum fatigue of women. 

We also found postpartum depression to be a major factor influencing women’s QoL. Previous studies have reported that postpartum depression reduces QoL in terms of physical and mental health [7,12,14,36]. Postpartum depression also tends to be exacerbated by postpartum fatigue or the burden of care for newborns in the early postpartum period, and it is highly likely to progress from mild depression to severe depression [30]. Lee and Cho [37] reported that there are more women with postpartum depression who have not been revealed given the Korean social prejudice to avoid psychiatric treatment. Therefore, postnatal care providers should detect and continuously manage postpartum depression based on accurate counting by integrating the screening for postpartum depression in postpartum management. In addition, given that the risk of severe depression increases when the support of the spouse or family is not satisfactory [26], healthcare providers should develop a strategy that can improve the relationship and intimacy between spouses [38] and enhance family support. For instance, interventions delivered through home visiting or training may reduce the risk of postpartum depression [39].

After childbirth, women need to quickly adapt to their new role as a mother: breastfeeding and caring for the baby, all while undergoing the physical changes related to lactation [40]. However, difficulty in lactation affects women’s QoL; women who fully breastfeed have reported higher QoL compared with women who practice mixed lactation [38]. Women who regularly breastfeed also have a higher degree of adaptation to breastfeeding compared with those who do not breastfeed regularly, which consequently lower postpartum fatigue and depression, ultimately improving their QoL during the postpartum period [14,37]. For effective breastfeeding adaptation, the participation of the husband in breastfeeding in the early postpartum period helps overcome the difficulties of breastfeeding for the mother as well as increases the interaction with the baby and the rate of breastfeeding practice [40]. In addition, the mothers who first attempt breastfeeding 1–6 h after delivery are more likely to adapt to breastfeeding than those who start after 24–48 h [37]. Therefore, prenatal breastfeeding education, in which the spouse participates, and continuous lactation management from the early postpartum period could contribute to the improvement of the QoL of postpartum women.

Among the general characteristics of the participants, occupation was found to be a factor affecting the QoL of postpartum women. Postpartum women with jobs have a high QoL as they can continue their social life and economic independence [13]. However, various studies have reported different results, and as such, a study that confirms the difference in postpartum women’s QoL in consideration of their job and income level is needed. In the current study, the number of the child was not found to be an influencing factor in the QoL of early postpartum women. These results are consistent in that the parity had no impact on postpartum QoL [41], unlike in studies where multiparity was a determining factor for lower QoL [42], or where nulliparity negatively affected postpartum QoL [43]. This could be a result of cultural differences targeting mothers within six weeks of staying at the postpartum care center in this study. Women at postpartum care centers in Korea receive overall postpartum care as well as breastfeeding management by health providers. In addition, the burden of having to take care of other children seems to be low; hence, such results came out.

This study had some limitations. First, the results should be generalized with caution because this study investigated women in a specific a region, and the subjects who participated in this study were healthy women with no complications related to pregnancy and no medical history. Second, as a cross-sectional study of only mothers within six weeks after childbirth, the findings were limited in terms of elucidating QoL throughout the early and late postpartum period. Additionally, we assessed marital intimacy using a questionnaire that encompassed all age groups of married women [24]. Future research should develop and apply instruments that reflect the characteristics of women during the postpartum period. 

Despite these limitations, this study is meaningful in that it identified the factors influencing QoL of women during the postpartum period from an integrated perspective and prepared fundamental data for intervention development to improve QoL.

## 5. Conclusions

This study identified the factors affecting the QoL of women during the postpartum period by examining the physical, psychological, and relationship aspects related to this period. To improve the QoL of women during the postpartum period, health care providers should develop interventions that can improve partners’ intimacy, reduce postpartum fatigue and postpartum depression, and help women adapt to breastfeeding. In addition, it is necessary to more attention to new mothers who do not have a job. Accordingly, such interventions would not only benefit women during the postpartum period but also improve the health and QoL of families, including the spouses and infants.

## Figures and Tables

**Table 1:** General Characteristics of the Participants (*N* = 179).

Variables	Categories	*n* (%)	M ± SD	Range
Age (year)	<35	116 (64.8)	32.9 ± 4.3	23–44
	≥35	63 (35.2)		
Age of spouse (year)	<35	74 (41.3)	35.7 ± 5.0	22–51
	≥35	105 (58.7)		
Education level	≤High school	33 (18.4)		
	≥College	146 (81.6)		
Job	Yes	77 (43.0)		
	No	102 (57.0)		
Religion	Yes	73 (40.8)		
	No	106 (59.2)		
Pregnancy type	Nature	164 (91.6)		
	Artificial	15 (8.4)		
Delivery type	Vaginal delivery	70 (39.1)		
	Cesarean section	109 (60.9)		
Baby’s gender	Male	99 (55.3)		
	Female	74 (41.3)		
	Mixed twins	6 (3.4)		
Baby’s birth order	1	99 (55.3)		
	2	66 (36.9)		
	≥3	14 (7.8)		
Baby care assistant	Yes	113 (63.1)		
	No	66 (36.9)		
Monthly household	<200	15 (8.4)		
income (KRW 10,000) ^a^	200–299	62 (34.6)		
	300–399	37 (20.7)		
	≥400	65 (36.3)		

Note: M = mean; SD = standard deviation. ^a^ KRW 10,000 = USD 9.02.

**Table 2:** Scores of Postpartum Fatigue, Postpartum Depression, Marital intimacy, Breastfeeding adaptation and Quality of life.

Variables	Possible Range	Min	Max	M ± SD	Skewness	Kurtosis
Postpartum fatigue	30–120	31.00	89.00	56.83 ± 12.64	0.27	−0.32
Postpartum depression	0–30	0.00	24.00	8.88 ± 5.02	0.42	−0.10
Marital intimacy	15–75	21.00	75.00	56.22 ± 10.74	−0.45	0.01
Breastfeeding adaptation	27–135	60.00	135.00	94.71 ± 13.28	0.02	0.06
Quality of life	0–30	7.09	28.33	19.64 ± 3.82	0.08	−0.11

Note: M = mean; SD = standard deviation.

**Table 3:** Maternal Postpartum Quality of Life according to General Characteristics.

Variables	Categories	*n* (%)	M ± SD	t/F (*p*)
Age (year)	<35	116 (64.8)	20.16 ± 3.63	2.51 (0.013)
	≥35	63 (35.2)	18.69 ± 4.01	
Age of spouse (year)	<35	74 (41.3)	20.31 ± 3.81	1.97 (0.051)
	≥35	105 (58.7)	19.18 ± 3.78	
Education level	≤High school	33 (18.4)	18.89 ± 4.41	−1.25 (0.212)
	≥College	146 (81.6)	19.81 ± 3.67	
Job	Yes	77 (43.0)	20.86 ± 3.38	3.83 (<0.001)
	No	102 (57.0)	18.73 ± 3.89	
Religion	Yes	73 (40.8)	20.24 ± 3.50	1.73 (0.085)
	No	106 (59.2)	19.24 ± 3.99	
Pregnancy type	Nature	164 (91.6)	19.82 ± 3.62	1.45 (0.169)
	Artificial	15 (8.4)	17.77 ± 5.38	
Delivery type	Vaginal delivery	70 (39.1)	19.73 ± 3.71	0.24 (0.813)
	Cesarean section	109 (60.9)	19.59 ± 3.91	
Baby gender	Male	99 (55.3)	19.38 ± 3.89	0.61 (0.543)
	Female	74 (41.3)	20.02 ± 3.35	
	Mixed twins	6 (3.4)	19.28 ± 7.49	
Number of child	1	99 (55.3)	19.50 ± 3.87	0.25 (0.777)
	2	66 (36.9)	19.73 ± 3.66	
	≥3	14 (7.8)	20.24 ± 4.42	
Baby care assistant	Yes	113 (63.1)	20.19 ± 3.80	2.53 (0.012)
	No	66 (36.9)	18.71 ± 3.71	
Monthly household	<200 ^a^	15 (8.4)	17.93 ± 5.25	4.65 (0.004) d > a *
income (KRW 10,000)	200–299 ^b^	62 (34.6)	19.48 ± 3.47	
	300–399 ^c^	37 (20.7)	18.48 ± 3.43	
	≥400 ^d^	65 (36.3)	20.86 ± 3.67	

Note. M = mean; SD = standard deviation; * Scheffé test; KRW 10,000 = USD 9.02.

**Table 4:** Correlations between Variables.

	Postpartum Fatigue	Postpartum Depression	Marital Intimacy	Breastfeeding Adaptation	Quality of Life
	r (*p*)				
Postpartum fatigue	1				
Postpartum depression	0.57 (<0.001)	1			
Marital intimacy	−0.23 (0.002)	−0.28 (<0.001)	1		
Breastfeeding adaptation	−0.34 (<0.001)	−0.32 (<0.001)	0.41 (<0.001)	1	
Quality of life	−0.48 (<0.001)	−0.53 (<0.001)	0.67 (<0.001)	0.49 (<0.001)	1

**Table 5:** Factors Influencing the Quality of Life of Subjects.

	Model 1					Model 2				
	B	β	t	*p*	95% CI	B	β	t	*p*	95% CI
(Constant)	16.08		15.93	<0.001	14.09–18.08	9.977		5.01	<0.001	6.05–13.91
Age (ref. ≥ 35)	1.861	0.233	3.28	0.001	0.74–2.98	0.547	0.069	1.41	0.160	−0.22–1.31
Job (ref. = no)	1.406	0.183	2.18	0.030	0.14–2.68	1.254	0.163	2.94	0.004	0.41–2.10
Postpartum fatigue						−0.058	−0.192	−3.40	0.001	−0.09–−0.02
Postpartum depression						−0.145	−0.190	−3.25	0.001	−0.23–−0.06
Marital intimacy						0.166	0.466	8.79	<0.001	0.13–0.20
Breastfeeding adaptation						0.047	0.163	3.09	0.002	0.02–0.08
adjusted R^2^ (∆adjusted R^2^)	0.147					0.636 (0.489)				
F (*p*)	6.12 (<0.001)					32.09 (<0.001)				

Note. CI, confidence interval; ref. = reference.

## Data Availability

The data presented in this study are available on request from the corresponding author. The data are not publicly available due to privacy.
